# Tunable Broadband Radiation Generated Via Ultrafast Laser Illumination of an Inductively Charged Superconducting Ring

**DOI:** 10.1038/srep18151

**Published:** 2015-12-11

**Authors:** John Bulmer, Thomas Bullard, Brian Dolasinski, John Murphy, Martin Sparkes, Krste Pangovski, William O’Neill, Peter Powers, Timothy Haugan

**Affiliations:** 1 Department of Materials Science and Metallurgy, University of Cambridge, UK; 2 Aerospace Systems Directorate, Air Force Research Laboratory, Wright-Patterson Air Force Base, Ohio, USA, 45433; 3 Physics Department, University of Dayton, 300 College Park, Dayton, Ohio, USA, 45469; 4 Institute for Manufacturing, Department of Engineering, University of Cambridge, UK, CB3 0FS

## Abstract

An electromagnetic transmitter typically consists of individual components such as a waveguide, antenna, power supply, and an oscillator. In this communication we circumvent complications associated with connecting these individual components and instead combine them into a non-traditional, photonic enabled, compact transmitter device for tunable, ultrawide band (UWB) radiation. This device is a centimeter scale, continuous, thin film superconducting ring supporting a persistent super-current. An ultrafast laser pulse (required) illuminates the ring (either at a point or uniformly around the ring) and perturbs the super-current by the de-pairing and recombination of Cooper pairs. This generates a microwave pulse where both ring and laser pulse geometry dictates the radiated spectrum’s shape. The transmitting device is self contained and completely isolated from conductive components that are observed to interfere with the generated signal. A rich spectrum is observed that extends beyond 30 GHz (equipment limited) and illustrates the complex super-current dynamics bridging optical, THz, and microwave wavelengths.

Ultra-high current density and transport without dissipation make superconductors a promising candidate material for electromagnetic transmitters. Individual transmitter components such as superconducting antennas and waveguides are particularly efficient, especially when electrically small^1^,^2^. As a transmitter’s power source, superconducting magnetic energy storage (SMES) stores energy with the perpetual circulation of a super-current^3^. This is particularly attractive to large, directed energy transmission systems because of their rapid response to peak power demands and high efficiency^4^. As a transmitter’s oscillator, thin film superconducting photo-switches are dissipation-less in the off-state, have pico-second switch times, and support large current densities. These switches are triggered by the illumination of an incident ultrafast laser pulse (typically tens to hundreds of femto-seconds) that splits Cooper Pair electrons into photo-excited quasi-particles. The splitting and subsequent recombination modulates the kinetic inductance and produces pico-second voltage transients without bolometric effects^5^,^6^.

Superconducting photo-switches integrated into standard transmission systems have been shown to generate ultrawide band (UWB) microwave radiation^7^. Integrating a superconducting photo-switch together with a superconducting antenna (typically millimeters in dimension or less) has been shown to generate THz UWB radiation^6^,^8^,^9^. In both of these cases, the power is provided by an external power supply directly wired to the antenna. By contrast, in this communication, we combine all the superconducting transmitter components–antenna, waveguide, SMES, and photo-switch– together into a single transmitter device for controllable GHz UWB radiation. This device consists of a continuous, thin film superconducting ring centimeters in diameter. After inductively establishing a super-current in the ring, we illuminate the ring with a single ultrafast laser pulse from an external laser. This triggers an electromagnetic pulse which we detect in the GHz regime in the far-field. Our findings suggest that the ring’s diameter and track width, as well as how we illuminate the ring (either at a point on the ring or the entire ring globally), dictates the received UWB spectrum generated by the ring after the ultrafast interaction.

This device is interesting in that it may alleviate complications associated with connecting separate transmitter components, which is problematic in UWB design. The possibility of tuning without traditional electrical components and metallic circuits is demonstrated by modification of the UWB spectrum through changing the laser pulse illumination pattern on the ring. Also of note, this is one of the first demonstrations of generating radiation from fast transients in DC circuits. Fast transients in direct current (DC) circuits is an active area of research, not addressed by lumped parameter models or antenna theory, where radiation and near-field effects may become significant to the circuit’s energy balance and must be fully taken into account. The general result of recent theoretical analysis derives an oscillatory circuit current that departs from lumped parameter behavior as it interacts with the reactive near field, potentially with higher radiation efficiency^10^,^11^. Another important aspect of the device is that it demonstrates radiation from explicit symmetry breaking. A recent paper^12^ highlighted the fact that electromagnetic radiation requires explicit symmetry breaking of an electric field and in their case was demonstrated experimentally with insulating dielectric resonator antennas. In our case, not just the antenna, but the entire electromagnetic system to include the power source starts in a symmetric state and is totally self-contained. The device is physically isolated from wires (such as temperature sensors) and other metallic components that were shown to distort the signal. The complete isolation of this device is believed to be a first demonstration and provides a unique radiator system to study. The explicit symmetry of the isolated ring is broken selectively and remotely by the ultrafast laser pulse.

We experimented with centimeter-scale superconducting rings (intrinsically doped yttrium barium copper oxide (YBCO)) supported on a sapphire substrate (Fig. 1a) with a single 120 fs ultrafast laser pulse illuminating the ring’s surface. A pulse of this duration is known to create pico-second voltage transients in thin film superconductors through the de-pairing and recombination of Cooper pairs^5^,^6^,^7^.

To generate a UWB pulse a super-current must first be established in the ring. Originally this was to be accomplished with the typical procedure of field cooling where the ring is cooled below its critical temperature in the presence of an external magnetic field. Once below the critical temperature, the magnetic field is removed. Field cooling however has the drawback that it requires cycling the temperature through the critical temperature. As a faster alternative we found we could establish a DC super-current by firing one ultrafast laser pulse at the ring while it is in the presence of an external magnetic field. The external field is generated by a removable solenoid that is temporarily placed around the ring and its cryogenic container. In the presence of the magnetic field the superconducting ring generates a screening current preventing a change in magnetic flux through its center. An ultrafast laser pulse temporarily interrupts the ring’s screening current and allows migration of the magnetic flux into the ring’s center. After removing the solenoid, magnetic flux is trapped in the ring and the persistent DC super-current is established. The magnitude of the current is primarily calculated from the law of Biot and Savart from measurements taken of the magnetic field at the center of the ring with a removable hall probe^13^,^14^,^15^.

After the super-current is generated and the solenoid is removed, energy is now indefinitely stored in the ring without loss. To generate radiation from this stored energy, a second ultrafast laser pulse illuminates the super-current either at a point (Fig. 1b) or uniformly around the ring (Fig. 1c) and this triggers the UWB microwave response. This signal is detected with far-field antennas and high speed oscilloscopes as described in the methodology section. We found that metallic components in the ring’s vicinity, such as temperature probes and their associated wires, modified the microwave response. Therefore anything conductive within a 450 mm radius from the ring was removed.

## Results

**Figure 2:**
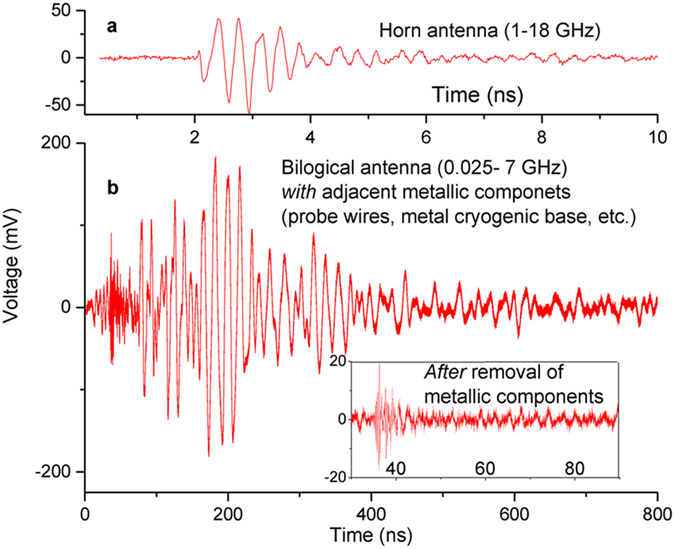
Representative, raw time domain signal from the 20 mm OD/5 mm track ring as recorded on the oscilloscope without any compensation for cable loss or the influence of the receiving antenna for (a) the primary horn antenna (1–18 GHz) placed in the plane of the ring. This shows the situation when the ring is isolated from metallic components. (**b**) The Bilogical antenna (0.025–7 GHz) without any cable or antenna compensation, placed 45 degrees above the ring’s plane. This signal is a result of an early experimental configuration where the ring was *not* isolated from objects such as metallic wires. Inset, the same receiving antenna configuration with metallic components removed.

Figure 2a shows a typical, raw time domain response as recorded by the oscilloscope. The typical signal lasts at least ~2 ns, approximately 17,000 times longer than the electromagnetic event that triggered it. The sustained, oscillatory nature of the voltage signal suggests a more complicated phenomena then a simple resistor/inductor current decay. The closest metallic surface was the optics table side ~450 mm away with a 3 ns round trip time from the ring. The signal beyond these initial 3 ns may be complicated by a mixture of multipath reflections; although frequency spectra analysis over the first 3 ns does seem to capture the bulk of the energy spectral distribution. Static magnetic field measurements with a Hall probe were taken in the ring’s center before the pulse to confirm the presence of the super-current. A number of measurements after the UWB response indicate ~2–10% of the field remains and suggests the entire superconducting ring did not go normal. Laser induced deterioration of the YBCO was occasionally a problem when focusing on a ring point with a liquid nitrogen film covering the surface. Otherwise, the YBCO rings robustly supported a super-current after many focused pulses.

Figure 2b shows a representative, raw signal in an early experimental configuration with metallic components adjacent to the ring. In these early experimental configurations the ring rested above a fixed inductive coil for energizing. Temperature and field probes were at one point fixed on the sapphire substrate with the probe wires extending well beyond the containment vessel. In another early configuration, the ring was not continuous, but had a break where electrical wires fed a DC current. All these early un-isolated cases generated a signal consisting of substantially longer wavelengths (bringing the receive antenna into the near field) and had a signal duration two orders of magnitude greater then what is seen in the isolated state (likely mixing the primary signal with multi-path reflections). Note that removing adjacent metallic components to include probes, their wires, and a metal based cryogenic system removed this long wavelength/long duration signal (Fig. 2b inset). Considering the primary interest here is the ring itself, we took care to isolate the ring from these metallic components and used a detachable Hall probe and a removable solenoid before triggering the UWB pulse. All analysis presented considers the ring in this isolated situation.

**Figure 3:**
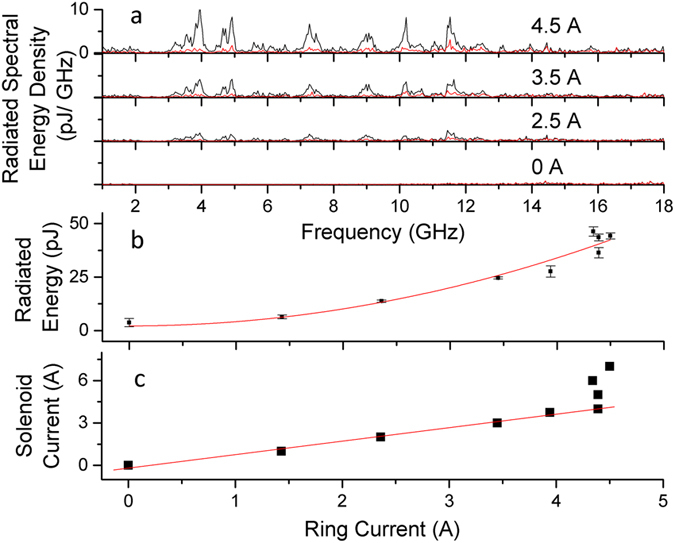
Radiation versus current for a 5 mm OD (outer diameter) with a 1 mm track and uniform illumination. (**a**) Evolving radiated spectral energy density growing with increasing ring current. Multiple measurements under identical conditions yield a standard deviation (shown in red). (**b**) Total radiated energy of the superconducting ring as a function of ring current with a quadratic fit. **c**, Linear relationship between the ring current and the removable solenoid current. This particular ring has a critical current at ~4.5 A.

Figure 3a shows the radiated spectra increasing in energy for increasing current in the ring. Integrating over each spectrum, Fig. 3b shows the total radiated energy at each ring current, which is fitted to a quadratic curve. This demonstrates the proportionality between the ring’s initial energy (which is proportional to the square of the ring current) and the radiated signal. Figure 3c shows the proportionality between the applied solenoid current and the ring’s current. This proportionality lasts up to the ring’s critical current, which for this ring is ~4.5 A at 77 K. Above this point, applying greater solenoid current leads to saturation in both the ring current and signal energy. Changing pulse energy and polarization changed the signal magnitude, but does not alter spectral shape (supplementary note 2). While a stronger laser intensity did not always generate a stronger microwave signal, a minimum intensity threshold did seem to appear at ~10% of the maximum. Note that intensity was adjusted by changing the laser pulse energy, not by adjusting the area of the spot size on the superconductor.

The effects of ultrafast laser pulse duration were investigated (supplementary note 3). For pulses shorter than ~500 fs neither the magnitude nor spectrum shape seemed affected. The maximum pulse length of ~1 ps however decreased the spectrum’s magnitude, but without changing the spectral distribution – an important practical consideration for implementing in compact systems. A 1 ns pulse of equivalent energy from another laser however did not produce the UWB response nor could it even discharge the ring. It has been shown that the interaction between the ultrafast pulse and the super-current creates a pico-second voltage spike from kinetic inductance modulation^5^,^6^,^7^. The fact that this photo-generated transient is expected to be ~1 ps may explain why the signal strength decreased when the laser pulse stretched to this value and why a 1 ns pulsed failed to produce any signal. Also from literature, it has been shown that with the relatively large laser intensities on the order of those used in this study, the typical ~1 ps voltage transient is then followed by a slower transient from bolometric effects which are possibly nanoseconds in duration^7^ .We note that this slower transient has a lifetime commensurate with the nanosecond duration of the detected signal. Considering that the triggered microwave response 1) changes when the ultrafast laser pulse extends beyond 1 ps and 2) requires these high laser pulse intensities, it is possible that both fast and slow transients are required to generate the UWB signals we observe.

**Figure 4:**
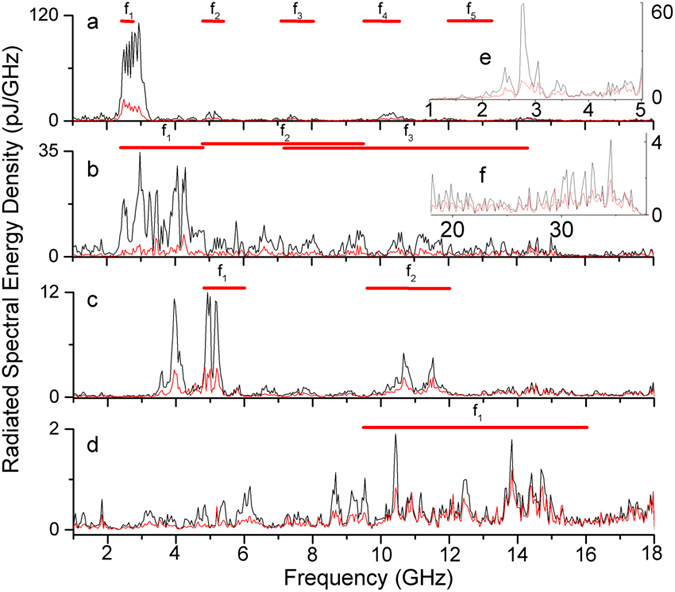
Radiated spectral energy density from point illumination. Red horizontal bars indicate the calculated harmonics (*f*_*n*_ = *n c*/*2d)* associated with travel around the ring’s circumference *d* and back, with the width of the bar corresponding to the width of the track. (**a**) 20 mm OD/1 mm track. (**b**) 20 mm OD/5 mm track. (**c**) 10 mm OD/1  mm track. (**d**) 5 mm OD/1 mm track. (**e**) (inset), the same ring in (**a**) but with a 45 ° above-plane measurement with another antenna. (**f**) (inset), the same ring in (**b**) but a spectra from a higher frequency antenna showing signal stretching to ~40 GHz. The oscilloscope has a roll-off at 30 GHz and signal beyond this should be considered qualitatively with respect to the noise floor.

Next we show the spectrum altering effects of different ring geometries and light placement. Figure 4 shows a representative data set chosen to demonstrate the effect of point illumination on rings of various dimensions, with radiation measured in the ring’s plane. Solid red horizontal bars above indicate the calculated integer *n* harmonic (*f*_*n*_ = *n c*/2*d*) associated with the speed of light *c* transit around the ring circumference, *d*, and back. Here we assume the impedance mismatch at the point of ultrafast illumination reflects the electromagnetic wave resulting in a round trip distance, 2*d*. As discussed earlier, at the laser intensity we used, it is very possible a ~ns duration “hot spot” formed after the initial ~ps transient leading to the ~ns duration signal. Such “hot spots” play a similar role reflecting electromagnetic radiation in intrinsic Josephson junction stacks^16^. A travel speed of *c* is justified provided the electric field connecting one part of the ring’s track to another is primarily contained in a medium with a dielectric constant equal to free space, which is potentially satisfied because the ring is enclosed in nitrogen gas. Indicated by the width of each red bar, each harmonic has a spread based on the ring’s non-zero track width.

As shown many of the peaks are consistent with this resonator model. The fundamental peak tends to have the largest amplitude, harmonics are spaced nearly in multiples of the fundamental, and the rings with widest tracks produce the widest peaks. In this model the peak widths increase for both larger track widths and for higher harmonics. For the ring with the largest track-width, the spectrum stretches to nearly 40 GHz (Fig. 4f). We also note that changing current or laser intensity only altered the signal magnitude and not the spectral distribution of the signal (see Supplementary note 2 for laser intensity results). In terms of this resonator model this demonstrates that neither laser intensity nor higher current levels excite higher harmonics, but only changes the magnitude of the response.

While this model captures many features in the energy spectra, not all peaks are covered and dispersion still needs to be addressed. Depending on the degree in which the electric field extends beyond the ring’s plane, the sapphire substrate below and nitrogen gas above would form an effective dielectric constant, likely frequency dependent^17^,^18^,^19^. Dispersion free behavior requires, at a minimum, transverse electric magnetic (TEM) modes of propagation^20^; full wave numerical simulation could shed light on the degree of feasibility in TEM modes in our system. Curvature in microstrip waveguide combined with a large track width is also a known source of dispersion^20^,^21^ and curvature in microstrip ring resonators affects the resonance frequencies of radial modes by changing the effective track width^19^,^22^,^23^. We note that the spread in our harmonic peaks are closely correlated with the ring’s track width, as indicated by the depicted red bars. The degree to which dispersion and curvature effects seen in these microstrip devices, which have a ground plane, can be extended to our experimental configuration, a stand-alone ring, is unclear. Further experimental investigation supplemented with modeling will help to elucidate these unknowns.

**Figure 5:**
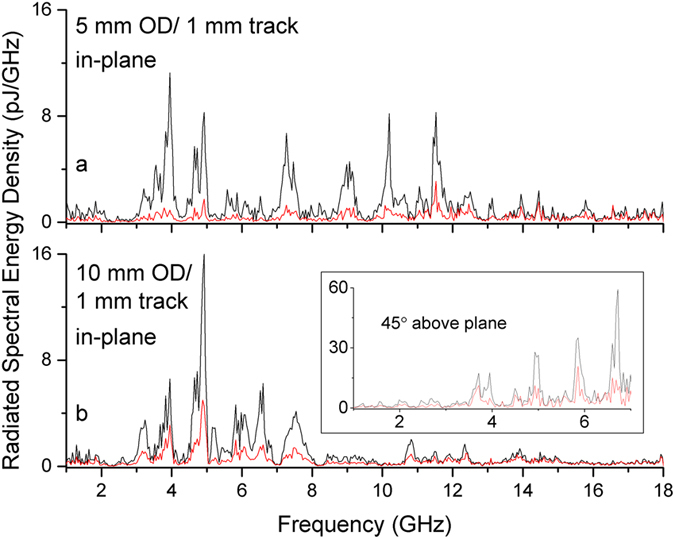
Radiated spectral energy density from uniform illumination. (**a**) 5 mm OD/1 mm track. (**b**) 10 mm OD/1 mm track. Inset, 10 mm OD/1 mm track measured 45 degrees out of plane.

We next consider uniform laser illumination. Here an intensity threshold only permitted successful triggering of the two smallest rings. Global illumination of the largest diameter did not generate detectable radiation. The measured spectrum from the ring with half the diameter appears to have twice the bandwidth. Harmonics are no longer multiples of any obvious fundamental, and the simple ring resonator model, where peaks relate to travel around a ring’s circumference, does not apply (Fig. 5). For radiation measured above plane, the 10 mm outer diameter (OD) ring shows resonant phenomena with higher frequency partials growing in intensity (Fig. 5 inset). The ultrafast laser pulse had a Gaussian spatial distribution, which means the rings receive a different intensity due to their different diameter. Intensity studies demonstrate that the difference in intensity only affects the spectrum magnitude and not the spectral energy distribution (supplementary note 2). Bandwidth corresponding with overall physical size is a natural result and similar size dependent effects have been seen with superconducting THz antennas^8^.

For both uniform and point illumination, spectra measured in-plane often corresponds to spectra measured out-of-plane. For example, Fig. 4e shows a 45 above-plane measurement with the same 3 GHz fundamental peak as the corresponding in-plane measurement (See supplementary note 4 for additional examples). In some circumstances however, above-plane measurements show additional spectrum structure or little correlation. The inset of Fig. 5 shows spectral content measured above-plane that, above 5 GHz, does not appear in the in-plane measurement. The observed angular dependence may correspond to behavior observed in loop antennas where the radiation pattern transitions from nominally in-plane to out-of -plane as the excitation current’s wavelength becomes equal to or smaller than the loop-antenna circumference^24^.

## Discussion

We demonstrated a transmitter that generates UWB GHz radiation from an ultrafast perturbation in a super-current with light placement and ring geometry controlling the spectrum. The radiation is not a result of a simple decay from a RL circuit, but rather frequency content in the microwave spectrum relate with the ring’s dimensions. With point illumination, a circumference resonator mechanism seems to capture many features of the data. Uniform illumination does not appear to fall under this model, but still has a bandwidth dependent on its physical dimension. Radiation is detected both in and out of the plane of the transmitting ring by two different antenna types, and in many cases the frequency content of the detected signals correspond with each other. Differences are explained by well documented phenomena observed in resonant loop antennas. This is an emerging concept and questions remain. For example, the degree to which illumination spot size affects the spectrum still needs to be investigated. Likewise, location of the spot on the ring relative to the detecting antenna and differences in the film between rings may also have an impact.

We observed that radiated energy scales linearly as a function of the ring’s initial energy, while the spectral distribution remains unchanged. Varying laser pulse intensity also does not change the spectral distribution demonstrating that both current and laser pulse intensity changes the amplitude, but not the frequency of the ring’s resonant response. We showed that the ultrafast pulse duration begins to decrease the microwave signal as it is stretched to ~1 ps, corresponding to the time scale of the pairing-depairing dynamics of the superconductor’s Cooper pairs. Again, the spectral distribution remained unchanged.

We believe these geometry dependent mechanisms are initiated by the ultrafast superconducting photo-response, but are not directly part of the THz spectrum associated with ~ps switching. In general the isolated peaks in the microwave spectrum contrasts with the broad THz spectrum generated by ~ps switching in sub-millimeter, photo-induced superconducting devices^6^,^8^,^9^. If the microwave spectrum were merely the tail end of a broader THz spectrum, we would expect to see a steady signal increase with frequency in both the 1–18 and 18–40 GHz measurements, but this was not observed. Due to the fact that this device relies on the interaction of an ultrafast laser pulse with a super-current, THz radiation is a likely by-product and will be investigated in later experiments.

For experimental purity, the experiment was accomplished with a single laser pulse on an electrically isolated ring. Important for applications however, we found continual transmission was straightforward with a kHz train of laser pulses and an externally applied, pulsed DC field from a fixed solenoid below. Generating GHz UWB radiation through ultrafast perturbations in a super-current induced in a superconducting thin film ring is an inherently compact and possibly efficient process that unites the power supply, waveguide, and transmitter together.

## Methods

Star Cryoelectronics manufactured the YBCO rings with a photolithography process from 330 nm thin film YBCO on R-plane sapphire wafer coated with 40 nm of CeO_2_ (critical current 3.4 MA cm^−2^, critical temperature 87.8 K). The ring was pasted with thermally conductive cryogenic epoxy onto a sapphire rod. The ring with sapphire rod was placed into a Styrofoam container, which was filled and maintained with liquid nitrogen up to the sapphire wafer that supported the ring. Nitrogen boil was sufficient to keep the ring moisture/frost free. The temperature was maintained at 77 K and was stable enough to generate consistent and repeatable measurements. The charging solenoid was 6.75 cm long and had 48 turns.

A Gaussian, linearly polarized, single ultrafast laser pulse came from an Spectra-Physics Hurricane Ti:Sapphire regenerative amplifier at 808 nm. Typically the energy per pulse was 1 mJ over 120 fs. Fluence ranged from 1–130 mJcm^−2^ depending if a ring spot was illuminated or an entire ring was uniformly illuminated.

The primary antenna was a horn antenna (with calibrated gain curve 1–18 GHz) placed 1.6 m from the ring (in the calculated far-field) in the plane of the ring. A Bilogical antenna (a biconic/log-periodic hybrid antenna with calibrated gain curve 25 MHz- 7 GHz) was placed 45 degrees from the plane of the ring, 1.9 m away. This antenna monitored for signal frequencies lower than the primary horn antenna, but it is understood that its full calibrated gain curve does not apply where wavelengths approach the transmitter/receiver separation. Being positioned at 45 degrees, this antenna monitored out-of-plane radiation. An optical mirror ~750 mm over the ring was necessary to direct the laser pulse and prevented placing an antenna directly over the ring. A third antenna (with calibrated gain curve 18–40 GHz) was sometimes used and was placed 1.4 m away from the ring. The antennas were connected to digitizing oscilloscopes (Lecroy 65 GHz LabMaster and Tektronix 33 GHz DPO73304DX) with characterized microwave cables. The sampling rate of the oscilloscopes satisfied the Nyquist condition for the detected pulse. The rise time of the signal was considerably slower than the oscilloscope’s minimum measurable rise time and fell within the bandwidth of the receiving horn antennas. Energy spectra referenced with respect to an isotropic radiator were calculated by applying the Friis equation and including gain and loss terms for the antenna and cable. Baseline signals of the laboratory’s electromagnetic environment were obtained and considered, which included microwave signals produced by the lasers electro-optic components (supplementary note 5). With as similar as possible cooling, power and laser conditions, the experimental sequence was executed typically three to ten times to develop a spectrum standard deviation.

The spectra shown are representative of the signal at the received horn antennas and, because the transmission transfer function is not yet known, the received signals only begin to indicate the rich current dynamics inside the ring. We assume the ring is an isotropic radiator as a standard reference to approximate the magnitude of the transmission. As shown with the out-of-plane antenna measurements, the transmitting transfer function of the ring will be a more complex spatial pattern. Note that other techniques to directly measure the ring’s current potentially involve placing conductive probes near the ring, but metallic components near the ring were shown to significantly alter the transmitted signal. The receive horn antennas were calibrated in the frequency domain using standardized procedures for a linear time invariant system and, considering the pulsed nature of the signal, a time domain calibration would initially seem more appropriate^25^,^26^. In this analysis however only energy magnitude versus frequency data was considered and additional phase information with the time domain calibration should not affect the magnitude information. Multipath reflections may contribute to the raw signal, but due to setup geometry would contribute only after the main pulse is captured by the receiving antenna. Removing, or “gating-out”, the time domain signal after this main pulse did not seem to affect the spectra significantly. Considering that all other experimental parameters were consistent between rings, the difference in spectra is best explained by differences in ring geometry and ultrafast laser pulse placement.

## Additional Information

**How to cite this article**: Bulmer, J. *et al.* Tunable Broadband Radiation Generated Via Ultrafast Laser Illumination of an Inductively Charged Superconducting Ring. *Sci. Rep.* **5**, 18151; doi: 10.1038/srep18151 (2015).

## Supplementary Material

Supplementary Information

## Figures and Tables

**Figure 1:**
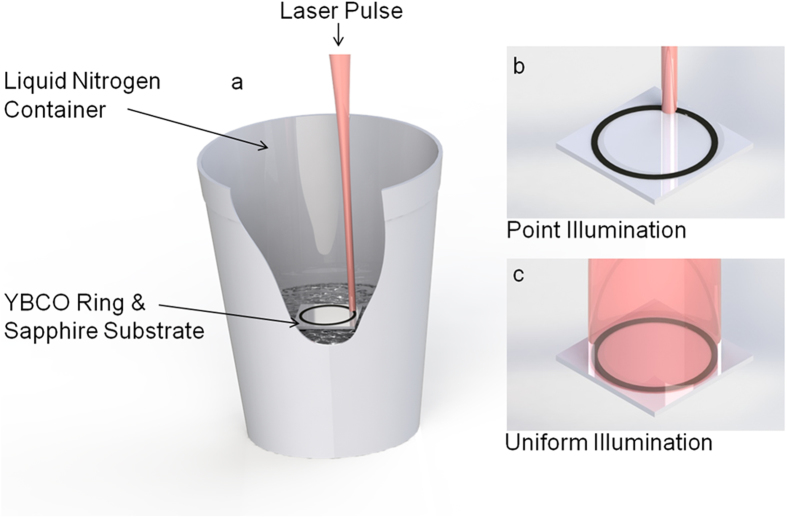
(**a**) Experimental centimeter-scale superconducting ring transmitter (no metallic components) with, (**b**) the single, ultrafast laser pulse illuminating the ring at a point and, (**c**) uniformly around the ring.
