# Supplementary material for: Tunable Broadband Radiation Generated Via Ultrafast Laser Illumination of an Inductively Charged Superconducting Ring
Source: Sci Rep. 2015 Dec 11;5:18151. doi: 10.1038/srep18151 (PMC4676057; doi:10.1038/srep18151)
Supplement: Supplementary Information [file srep18151-s1.doc]

**Supplemental Information For**

**Tunable Broadband Radiation Generated Via Ultrafast Laser Illumination of an Inductively Charged Superconducting Ring**

John Bulmera[[1]](#footnote-2), Thomas Bullard^b^ , Brian Dolasinski^c^, John Murphy^b^, Martin Sparkes^d^, Krste Pangovski^d^, William O’Neill^d^, Peter Powers^c^, Timothy Haugan^b^

a Department of Materials Science and Metallurgy, University of Cambridge, UK

b Aerospace Systems Directorate, Air Force Research Laboratory, Wright-Patterson Air Force Base,

Ohio, USA, 45433

c Physics Department, University of Dayton, 300 College Park, Dayton, Ohio, USA, 45469

d Institute for Manufacturing, Department of Engineering, University of Cambridge, UK, CB3 0FS

**Supplementary Note 1:** The following figures demonstrate the effects of ring size and illumination condition (either point or full) on the spectrum and total radiated energy.

**
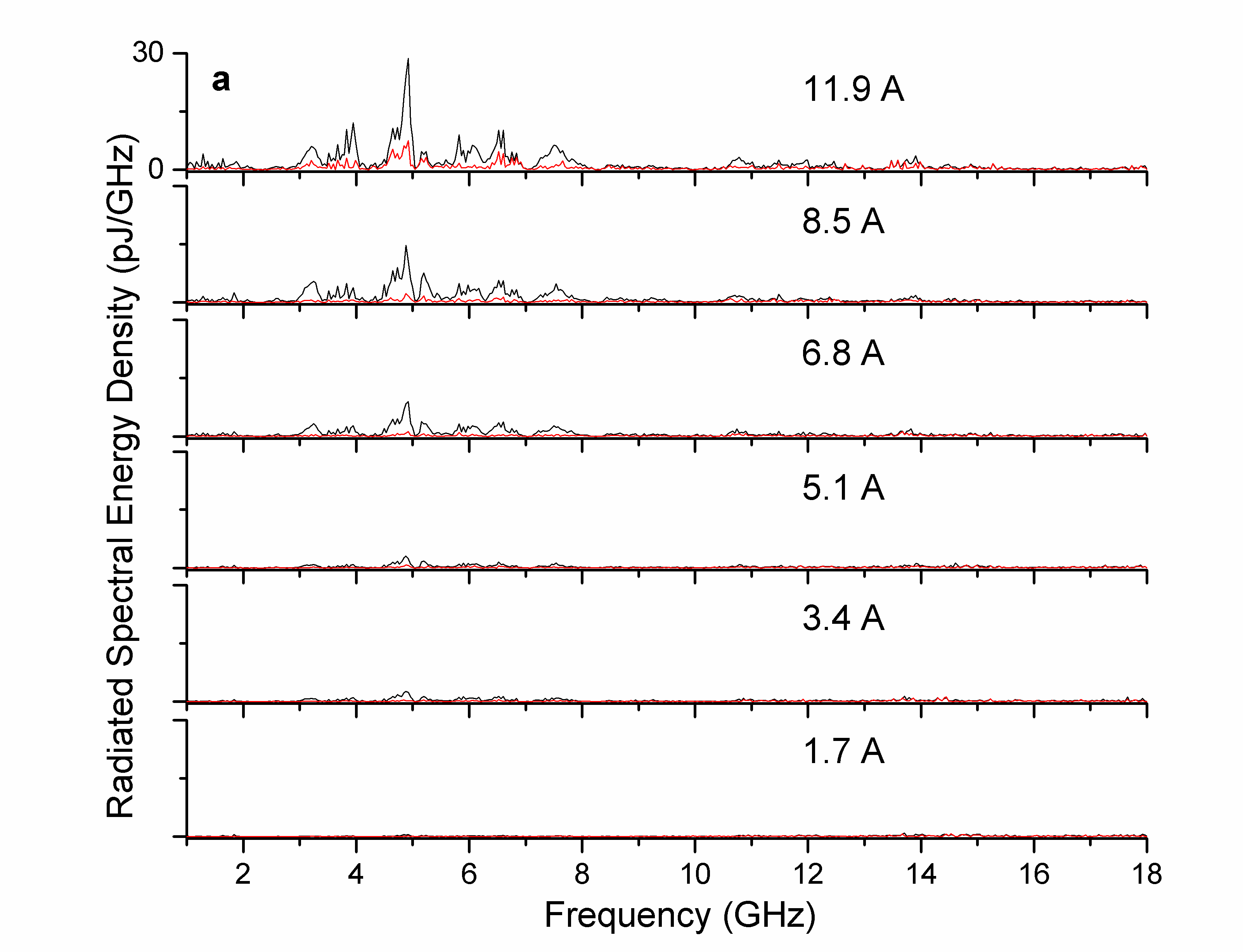
**

**
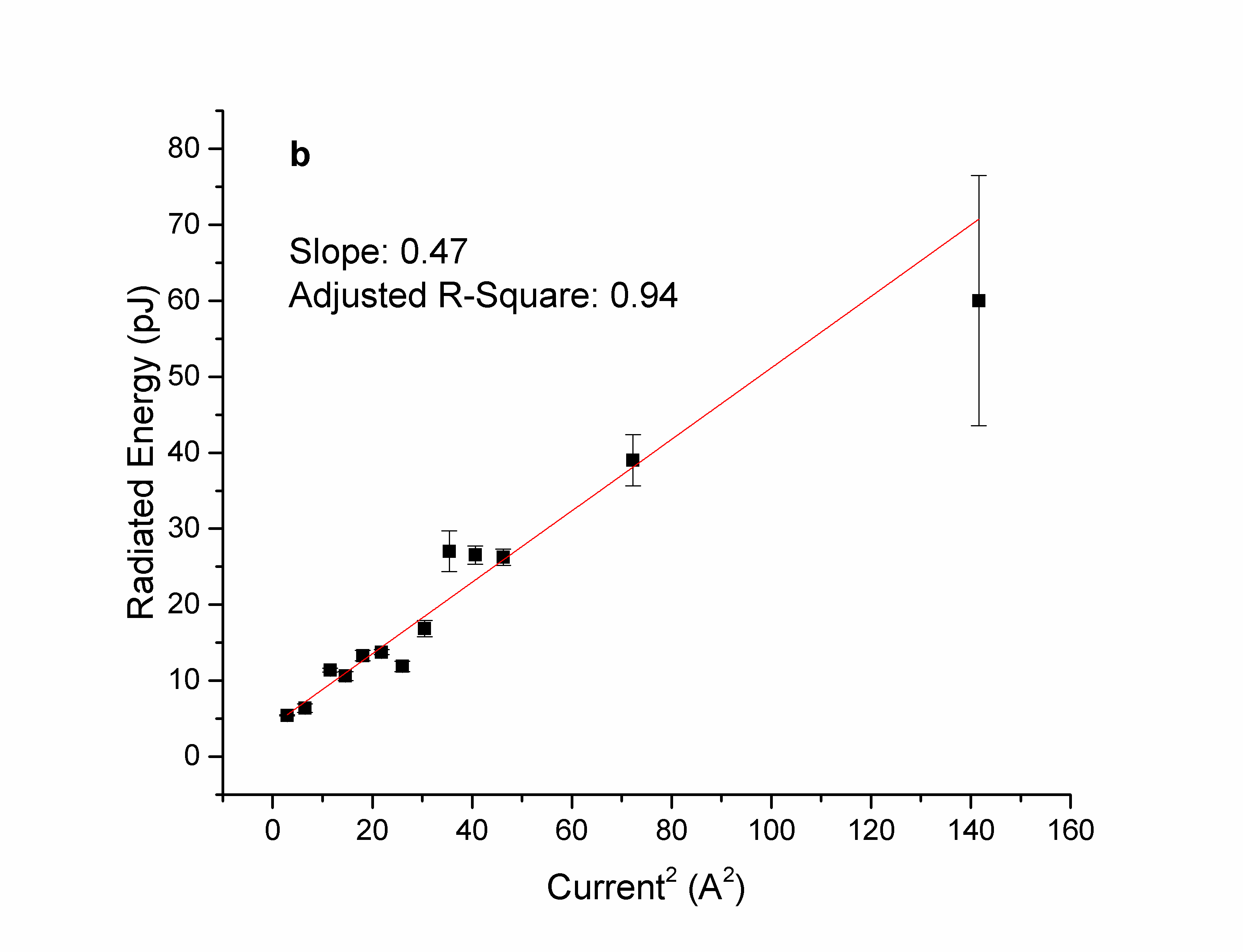
**

**Figure 1|** Current dependence for the case of 10 mm OD ring/ 1 mm track thickness, uniformly illuminated. **a**, Spectral energy density and **b,** total radiated energy both as a function of ring current.

**
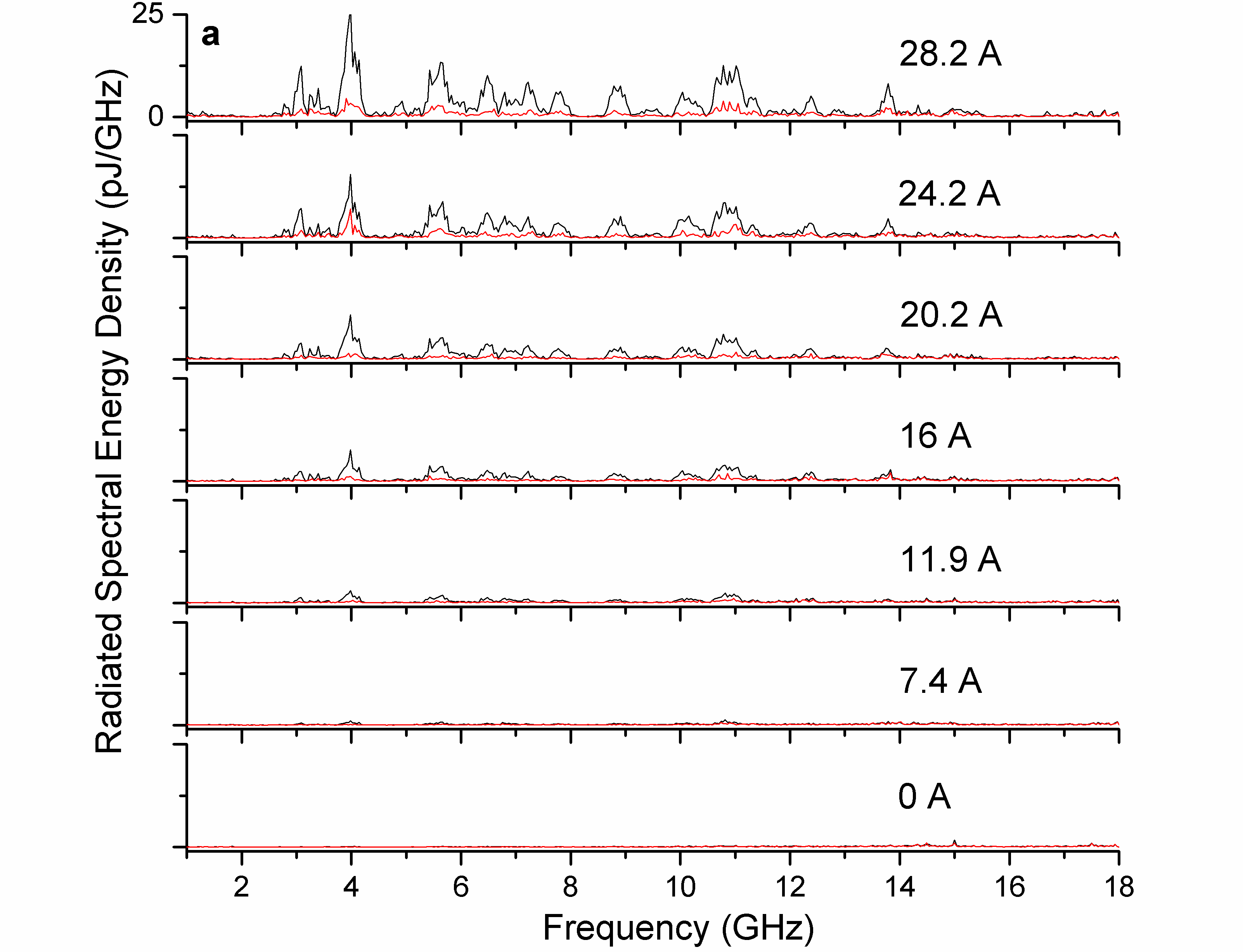
**

**
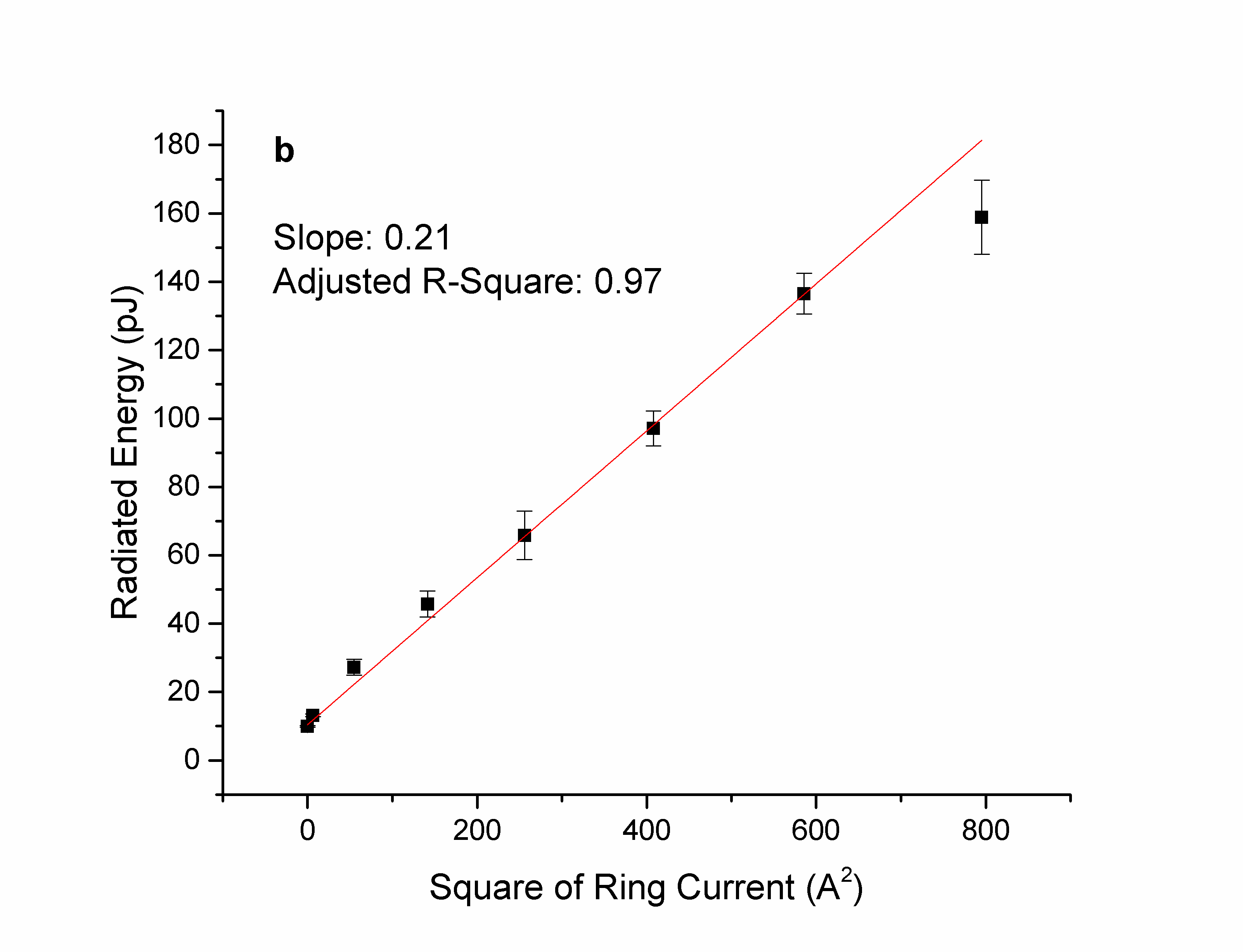
**

**Figure 2|** Current dependence for the case of 20 mm OD ring, 5 mm track thickness, illuminated at a point., **a**, spectral energy density and, **b**, total radiated energy as a function of ring current squared.

**Supplementary Note 2.** In the following example, laser pulse intensity affects the magnitude of the spectrum, not the energy spectral distribution. The area of the illumination spot and pulse duration remains the same; the intensity is change by increasing energy per pulse. The dependence of the spectrum’s magnitude on intensity is not linear, and sometimes a smaller intensity will yield a stronger signal. In general we observed that below a certain beam intensity the signal was undetectable. While a single pulse illuminates our samples, laser intensity was measured as a pulse train and not in single pulse mode. As a result, intensities are reported here as an average power in Watts.

**
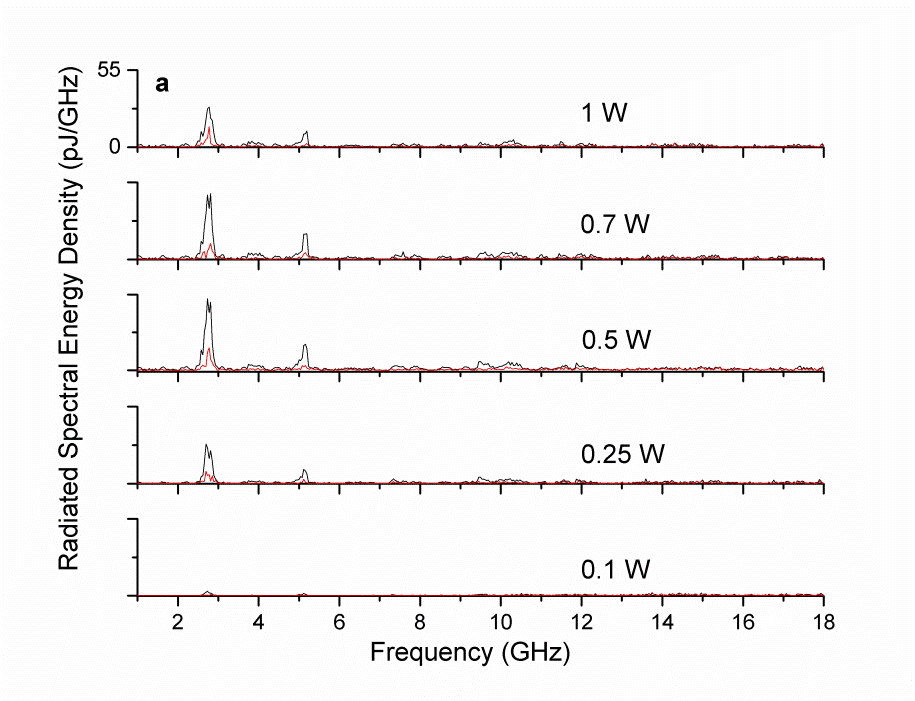
**

**
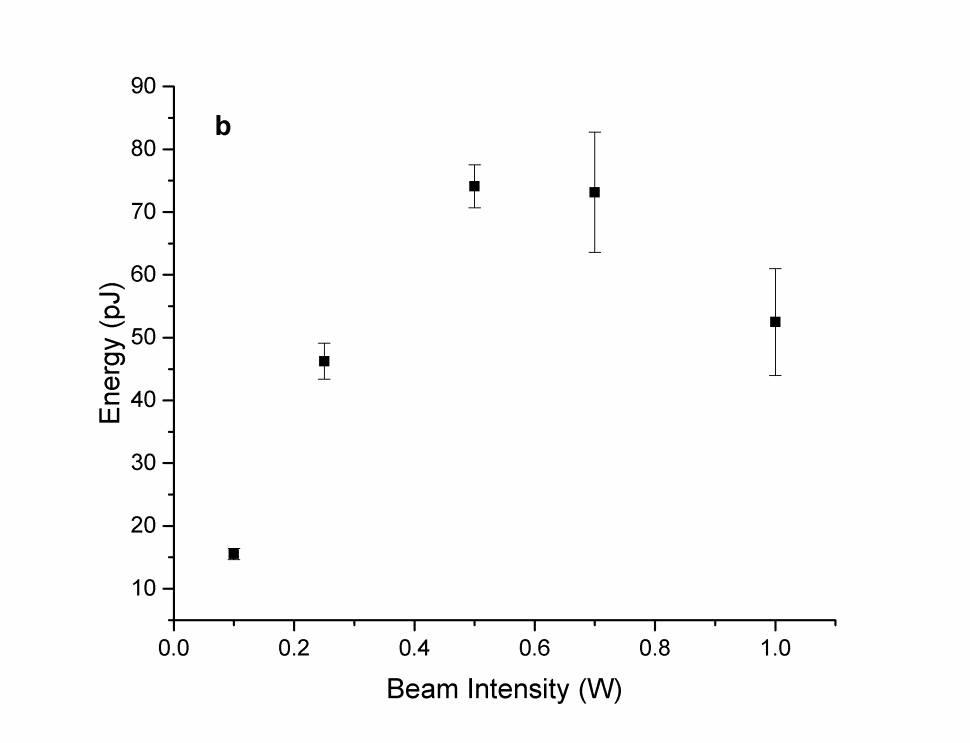
**

**Figure 1|** Changing laser intensity (point illumination) on the 20 mm OD/ 1 mm track ring. **a**, Spectral energy density and **b,** Total radiated energy as a function of intensity.

**Supplementary Note 3.** The grating compressor was adjusted to stretch the pulse duration. Pulse times given below are approximate. Stretching the pulse did not have an effect until after 300 fs, where at 1 ps the signal magnitude lowered. Spectral shape was unchanged as shown in figure 1.


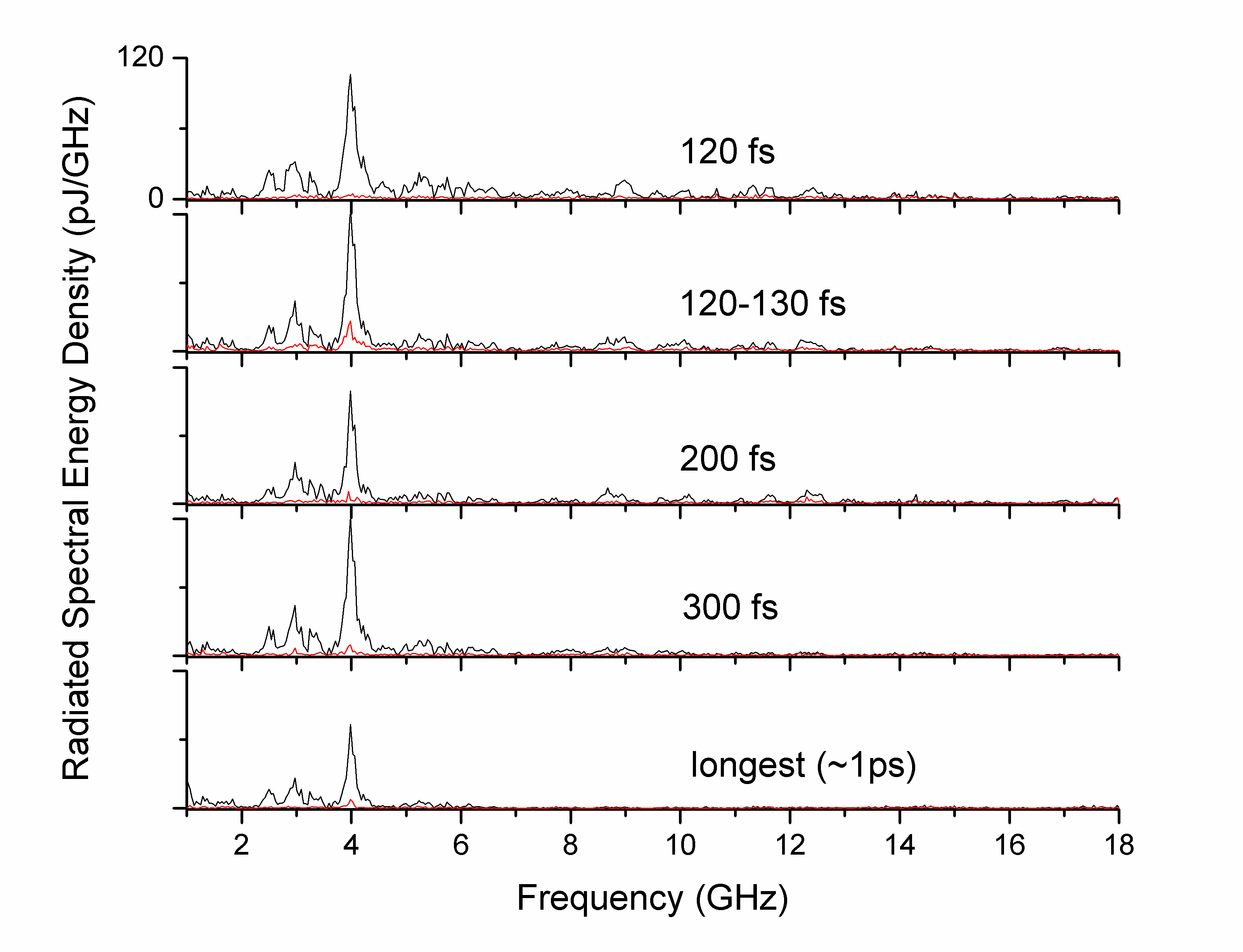


**Figure 1.** Effect of increasing the pulse time for a 20 mm OD/ 5 mm track ring. The track is illuminated at a spot on the outer edge of the ring and did not illuminate the full track. The energy output does not noticeably decrease until the pulse length reaches 1 ps. Spectral distribution remains essentially unchanged.

**Supplementary Note 4.** In the following examples the energy spectra are compared between radiation received in the ring’s plane and 45 degrees above plane. The primary receiving antenna was a horn antenna (1-18 GHz) placed in the plane of the ring. A Bilogical antenna (0.025-7 GHz) was positioned 45 degrees above the plane and in-line with the horn antenna. The purpose of the Bilogical antenna was to monitor for the existence of lower frequency signals, serve as redundancy for the primary antenna, and detect signals above the plane of the ring (not captured by the horn antenna).

We expected that the strongest radiation would be in the plane of the ring. However, it is well documented that the radiation pattern transfers from parallel to almost perpendicular the loop antenna’s plane when the wavelength of the excitation current approaches the loop’s circumference [1]. The Bilogical antenna was positioned to capture some of these signals.

The antenna could not be placed directly over the ring due to the equipment configuration in the lab. Therefore the Bilogical antenna was positioned at a 45 degree angle to the plane of the ring where it had an unimpeded line of site. The following figures compare the differences between the in-plane and above-plane measurements for the various ring and light geometries.

1. Chatterjee, M. Antenna Theory and Practice. New Age international Ltd. New Delhi, India (2004).


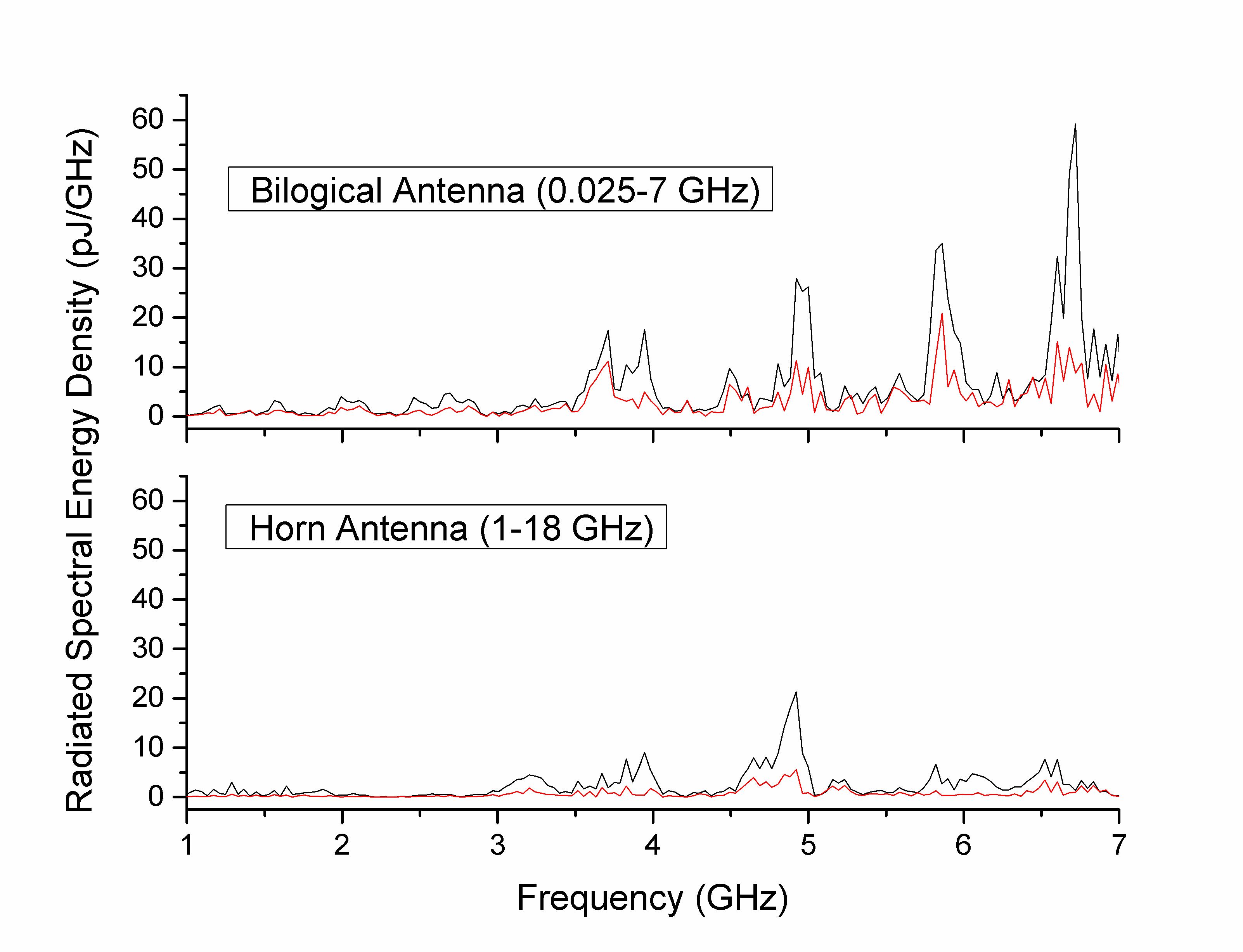


**Figure 1|** Comparison of the spectral energy distribution for the 10 mm OD/ 1 mm track ring, uniform illumination (Bilogical at 45 degrees above plane and the horn in the plane). Similar peaks are observed at 4 and 5 GHz while a definite difference in spectral content is observed primarily above 5 GHz.


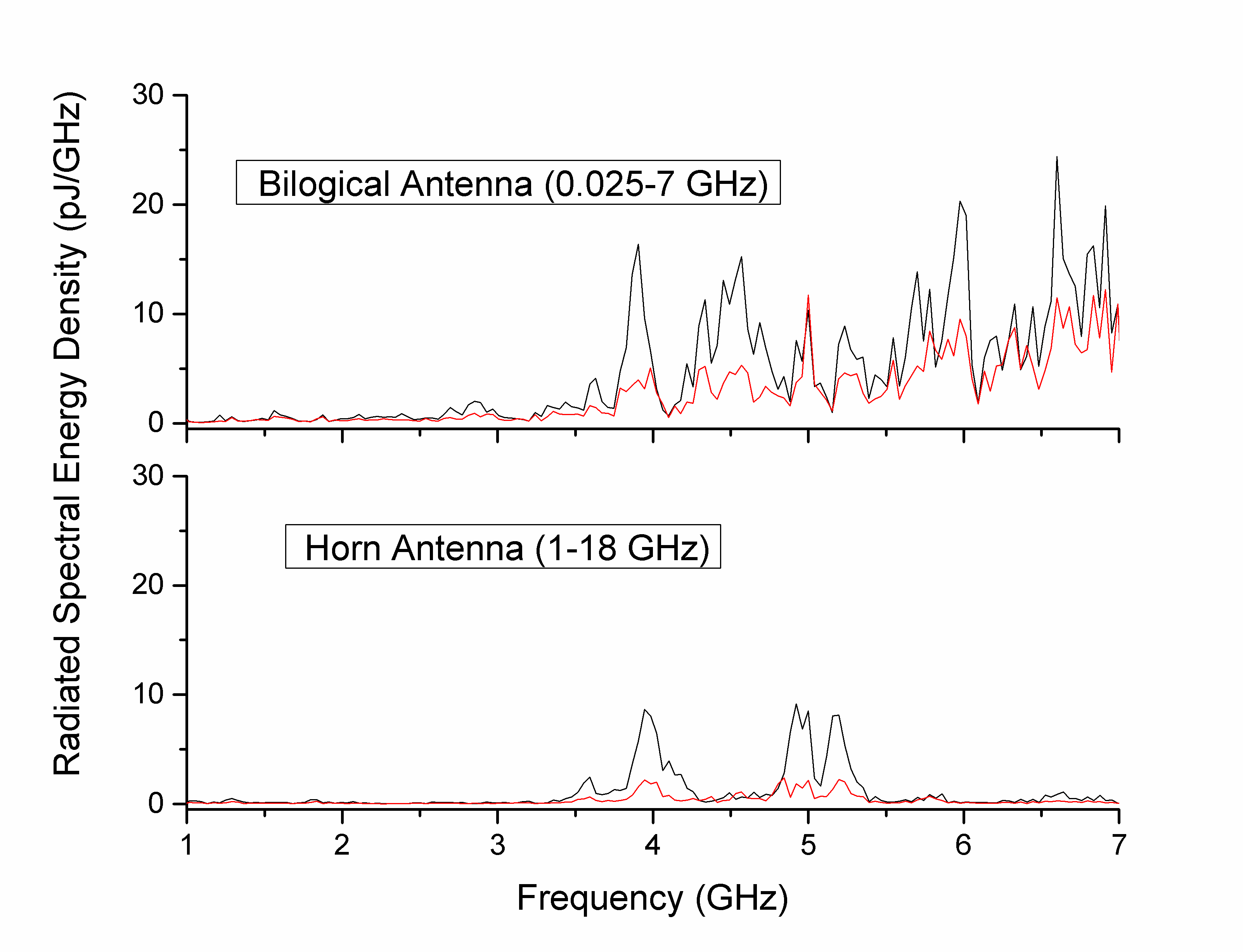


**Figure 2|** Energy spectra comparison for a 10 mm OD, 1mm track, point illumination (Bilogical at 45 degrees above plane and the horn in the plane). For the Bilogical antenna, the signal above 4.5 GHz is close to the noise floor.


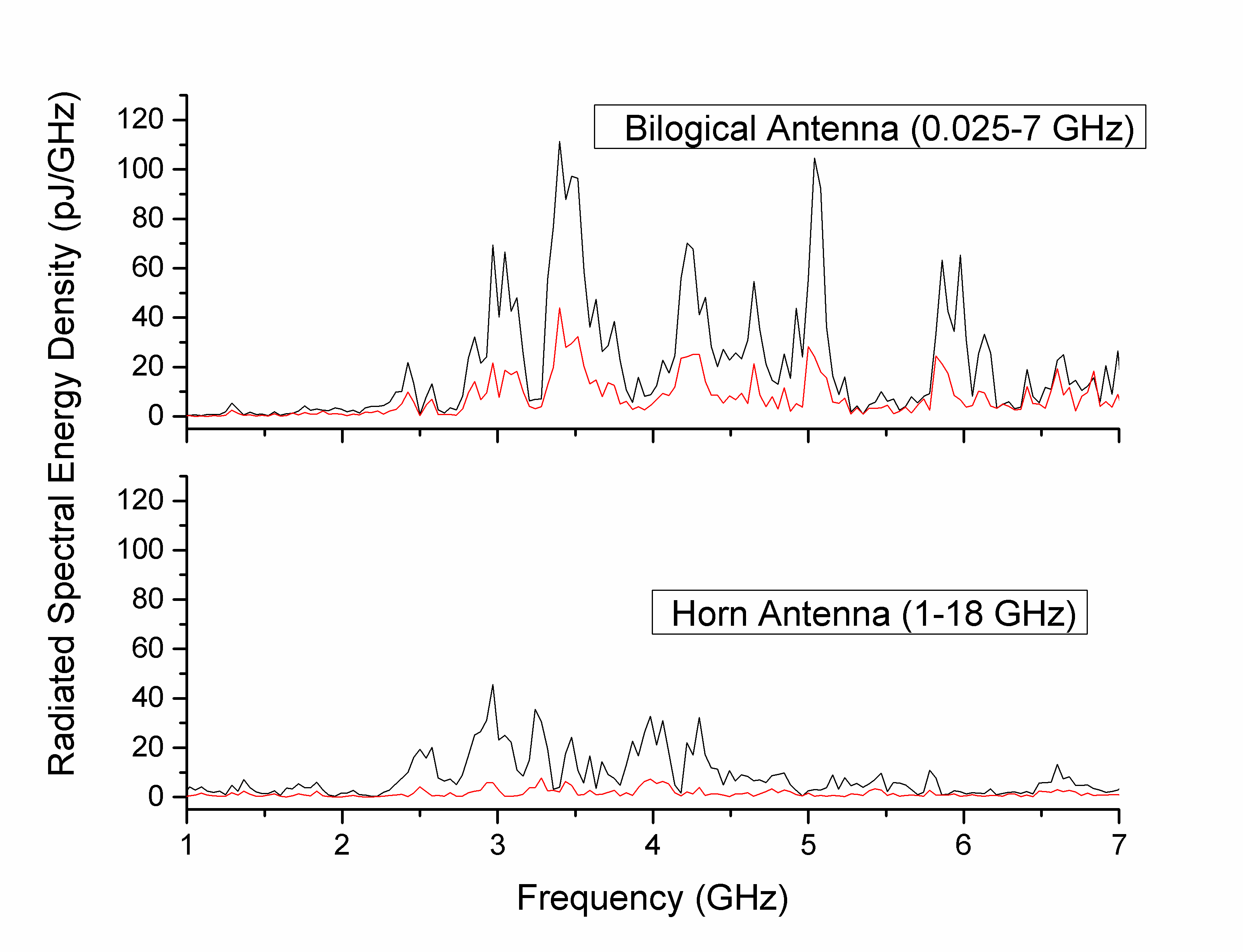


**Figure 3|** Spectral energy density comparison for the 20 mm OD ring, 5 mm track, point illumination with the illumination point covering the full track (Bilogical at 45 degrees above plane and the horn in the plane).

**Supplementary Note 5:** Following are represenative background signals from the antennas as shown in figures 1 through 3.

**
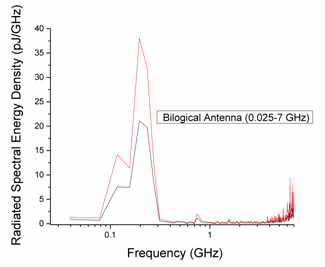
**

**Figure 1|** A logarithmic plot of the background from the Bilogical antenna, which was placed 45 degrees above plane of the ring. There is a large component at 200 MHz which was from electrical equipment in the laboratory.

**
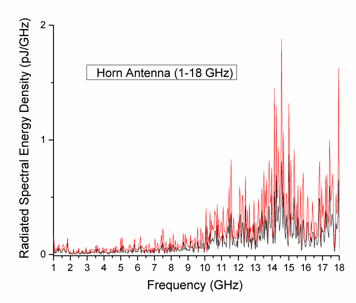
**

**Figure 2|** The background from the 1-18 GHz horn antenna positioned in the plane of the ring.

**
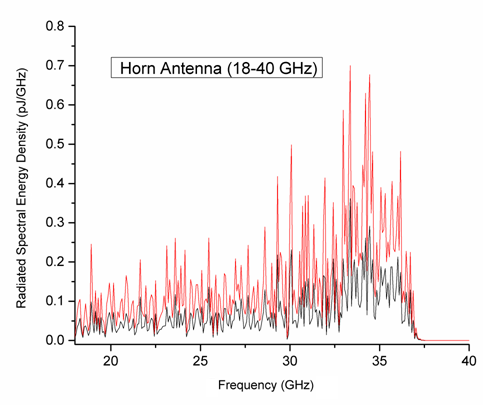
**

**Figure 3|** Background for the 18 to 40 GHz horn antenna (in the plane of the ring).

1. To whom correspondence should be addressed [↑](#footnote-ref-2)
